# Climate Change Vulnerability Assessment in Mangrove-Dependent Communities of Manoka Island, Littoral Region of Cameroon

**DOI:** 10.1155/2022/7546519

**Published:** 2022-09-05

**Authors:** Evariste Fongnzossie, Denis Jean Sonwa, Philippes Mbevo, Fabrice Kentatchime, Aurelie Mokam, Claude Tatuebu Tagne, Lydie Flora Essamba A. Rim

**Affiliations:** ^1^Advanced Teachers' Training College for Technical Education, The University of Douala, Cameroon; ^2^Center for International Forestry Research (CIFOR), Yaoundé, Cameroon; ^3^Department of Geography, Faculty of Arts, Letters and Social Sciences, The University of Yaoundé 1, Yaoundé, Cameroon; ^4^IBAY SUP—HIES, High Institute of Environmental Sciences, Yaoundé, Cameroon

## Abstract

This study was conducted on Manoka Island (Littoral Region of Cameroon) with the aim of analyzing climate change vulnerability and local adaptation strategies based on the local community's perceptions and biophysical evidence. We used household surveys, focus group discussions, field observation, GIS, and remote sensing to collect data on variables of exposure, sensitivity, and adaptive capacity. Historical changes in rainfall and temperature, mangrove cover, and the occurrence of extreme climatic events were used as indicators of exposure. Property losses and income structure were used as indicators of sensitivity, while human, natural, social, financial, and physical assets represented adaptive capacity. 89 households were interviewed in the nine settlements of the island. Results show that Manoka Island is experiencing irregular rainfall patterns (with average annual values deviating from the mean by −1.9 to +1.8 mm) and increasing temperature (with annual values deviating from the mean by −1.2 to +3.12). The dynamics of the coastline between 1975 and 2017 using EPR show average setbacks of more than ±3 m/year, with erosion levels varying depending on the period and location. The number of households perceiving extreme climatic events like seasonal variability, flood, and rain storm was higher. From respondents' perception, housing and health are the sectors most affected by climate change. The reported high dependence of households on fishing for income, their overall low livelihood diversification, and their poor access to climate information reported by 65% of respondents portray their poor adaptive capacity. Local response initiatives are ineffective and include among others constructing buildings on stilts and using car wheels to counter the advancement of seawater inland. The study concludes that households on Manoka Island are vulnerable to the impacts of climate change. Income diversification, mangrove reforestation, the development of sustainable supply chains for wood fuel, and sustainable fish smoking devices are the main pathways for adaptation planning in this area.

## 1. Introduction

Climate change has become a matter of concern in the current debates over environmental management across the world. The frequency and magnitude of extreme weather events, as well as sea levels, are expected to increase [1, 2]. The ecosystems that are most sensitive to all these disturbances are those of forests, mountain environments, and wetlands including mangroves, which represent coastal ecosystems bordering tropical seas [3].

In coastal areas in particular, previous studies identified some potential biogeophysical hazards to which coastal areas are exposed, including floods, strong winds, coastal erosion, increased salinity of estuaries, alteration of the quality of water available in aquifers, and the disappearance of certain “wet” zones and low-lying coastal fringes [4]. Storms affecting daily life and the economy in marine and coastal areas were also pointed out by some researchers [5], calling for better knowledge about the vulnerability and mitigation or adaptation measures that reduce their impact.

Coastal areas have an invaluable ecological, economic, and social role to play at both local and global levels [6, 7]. The climate-related threats they face can have negative effects at the socioeconomic and environmental levels by acting specifically on species distribution [8], agriculture, drinking water supply, health systems, and mangroves. According to the sixth report of the Intergovernmental Panel on Climate Change (IPCC) [1], the global temperature was 1.09°C higher in 2011–2020 than in 1850–1900 and the sea level rose by 3.7 mm/year between 2006 and 2018, with human influence being the likely main driver since at least 1971. According to the World Meteorological Organization (WMO) [2], in much of Africa, the temperature has already risen by more than 1°C since 1901, with increased numbers of heat waves and scorching days. This increase in temperature and changes in rainfall patterns also significantly affect population health across Africa.

Worldwide, scientists have studied and helped improve our understanding of mangroves in the context of a warming climate, emphasizing their vulnerability and also knowledge gaps around climate change impacts [9]. Historically, mangrove areas in Africa, which accounted for 20% of world mangrove areas in 2001, have changed in response to sea-level rise, resulting from the thermal expansion of ocean water and melting of the polar ice caps [9].

In Cameroon, mangroves are particularly threatened by various anthropogenic activities (fish smoking, construction, sand extraction, population growth, urban infrastructure development, economic pressure from oil exploration, etc.), which have contributed to about 30% loss between 1980 and 2006 [10, 11]. Concerning the mangroves of the Wouri estuary, studies underlined that a strong anthropization is at the origin of many environmental impacts [12]. Based on an analysis of the spatial evolution of the Mabe Mangrove Reserve, an overall decrease of 5710.83 ha (13% decrease) between 1986 and 2014 corresponding to an annual loss of 1162.25 ha (0.48% decrease) of mangrove was reported [13]. The highest rate of degradation was between 1986 and 2000. This loss reflects the pressure on this ecosystem. Fuelwood collection is known to be one of the main drivers of mangrove degradation and deforestation [11, 14, 15]). A study conducted in the Douala-Edéa Reserve identified a total area of approximately 11,025.3 ha (or 4%) of completely degraded mangroves requiring reforestation [16]. A similar trend in mangrove forest degradation was reported for Manoka Island, where evidence showed a reduction of mangrove forest cover from 6459 ha in 1986 to 5665.59 ha in 2018 (14% loss) [15].

Cameroon national policy documents such as the National Communication on Climate Change and the National Adaptation Plan have regularly mentioned the Far North and coastal areas as the most vulnerable areas of Cameroon.

So far, some attempts have been made to understand the impact of climate change and adaptation strategies in the coastal areas of Cameroon.

Along the southwest coast of Cameroon, mudslides, high tides, storm surges, saltwater intrusions, flash floods, rainstorms, landslides and lava flows resulting from an eruption, wind storms, and strong erosion were reported as the main natural hazards [17, 18]. These natural hazards have caused damage to houses, classrooms, farmland, and cars and the loss of household appliances; in addition, people have been injured or lost their lives [17, 18]. This means there is a high vulnerability of the populations living there, particularly to flooding. In this area, studies reported that flood-triggered migration has meant the relocation of settlements to around 3.5 km inland over the past 45 years, with a corresponding loss of around 989 ha of mangrove forest cover [19]. Similarly, the relocation of more than 1286 people has been reported in Cape Cameroon following coastal erosion that caused significant coastline movement. Damage to the houses, total loss of houses, loss of farmland, loss of domestic animals, loss of agricultural crops, and landscape distortion were additional local effects [20, 21].

The vulnerability of Cameroon's mangrove ecosystems in the Wouri estuary has been investigated. While the mangroves of the Douala estuary in Cameroon have an overall resilience, some inherent vulnerability due to the low tidal amplitude of the area has been reported [22]. There is evidence of increased vulnerability due to decreasing rainfall and irregular rainfall patterns, increased occurrence of extreme climatic events, and increased levels of coastal erosion, resulting in several effects, including low adaptive capacity [23].

The synergy between adaptation and mitigation was pointed out as a way to promote resilient responses. In coastal areas, exploring adaptation options requires an assessment and understanding of existing vulnerabilities.

Manoka is a small tropical island town located in the Littoral Region of Cameroon. The island is situated about 50 km from the mainland area near Douala city. It is a periurban area with landscape development under the influence of urbanization. Different types of pressure are exerted on the natural resources of this fragile island, particularly on mangroves. There is currently little knowledge available on existing or future vulnerabilities on this island. Some previous vulnerability assessments have been conducted in the Littoral Region of Cameroon, but very few have quantitatively and holistically measured the vulnerability level of the whole social-ecological system with analysis of the spatial differences in Manoka, and this is the problem this study is aiming to address.

With the current advances in human understanding of vulnerability, this concept has evolved over time to integrate nature, economy, society, humanities, environment, and other comprehensive categories. In this study, vulnerability to climate change is understood as the extent to which a system is unable to cope with the adverse effects of climate change and climate variability [1]. The process of reducing vulnerability requires a thorough understanding of who is vulnerable and why. This work seeks to answer the following specific questions:

- How vulnerable are local communities on Manoka Island to climatic hazards?
- What are the underlying causes of their vulnerability?
- How do they respond to the perceived changes?

This study aims to assess climate change vulnerability (through its variables of exposure, sensitivity, and adaptive capacity) and local response strategies on Manoka Island.

This profiling of the vulnerability to climate change in this area will be important in developing appropriate adaptation strategies. The study is important as it will generate information that can be used locally (e.g., at the council level or at the subdivisional level) as well as nationally for better planning of a climate change response.

## 2. Materials and Methods

### 2.1. Study Area

**Figure 1:**
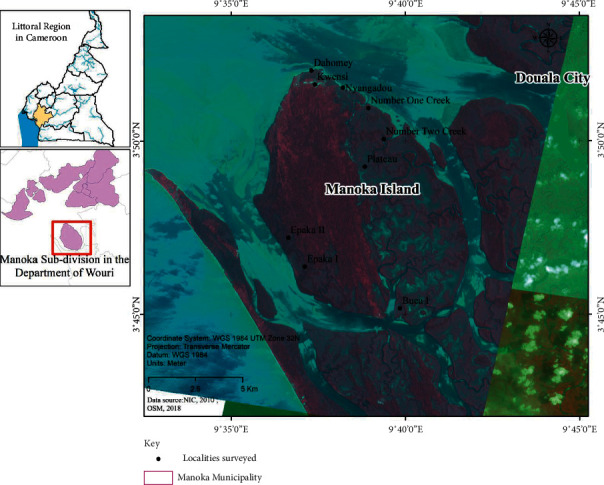
Location of Manoka Island and study settlements.

Manoka is a small island town located in the Littoral Region of Cameroon at 3°47′27.69″N and 9°36′45.78″E (Figure 1) and is the headquarters of the newly created Douala 6 subdivision. Manoka Island has a surface area of 10,031 ha and has a population of about 3371, consisting of 10% Cameroonians and 90% foreigners from West Africa [23]. Fishing is the main activity practiced by men and women of the island. Manoka Island is one of 24 islands found in this council area.

**Figure 2:**
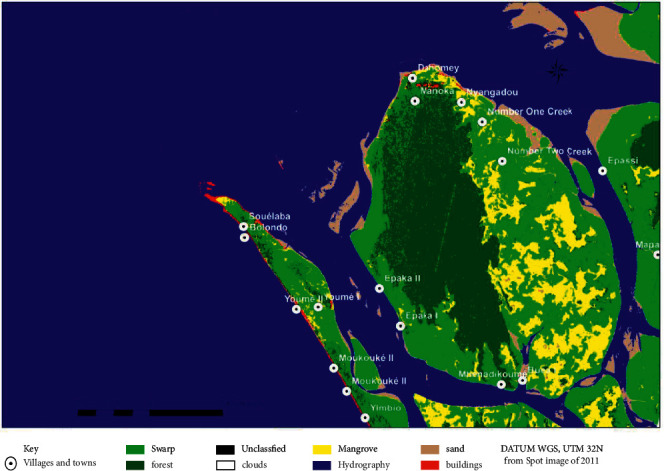
Land cover map of Manoka Island.

The average elevation is 12 m. Two types of ecosystems are present: mangrove and low-altitude coastal forest. To enhance the protection of the important ecosystems of this island, 55% of its surface area was classified as part of the Douala-Edéa National Park. A community forest (30% of the island) was also awarded to local populations. The remaining part of the island is referred to as community land where the population can do their activities. Several types of ecotones can be found on Manoka Island. These include the land-sea ecotone and the mangrove-land forest ecotone (Figure 2). With the increase of population, important parts of the island have gradually been transformed to form settlements [15].

The climate is of the equatorial type and is characterized by very high humidity of around 85%, especially in the rainy season; low thermal amplitudes that are not very variable depending on the location, with an average annual temperature of 27.2°C; and heavy rainfall, varying from 2400 to 4000 mm per year [20]. About 759 households exist in the area, making a total population of approximately 3371. The island is surrounded by the ocean and located not far from the Wouri River and Dibamba River. The island is thus under the influence of both continental and sea waters. Hydrologic, climatic, and meteorological factors related to coastal islands are thus impacting Manoka.

### 2.2. Vulnerability Assessment Framework

This study built on IPCC guidelines for vulnerability assessment [24]. The methodological framework was adapted from several assessment frameworks, one of which is the multidimensional framework for exploring the vulnerability of socioecological systems. It considers vulnerability as a function of exposure, sensitivity, and adaptive capacity [25, 26].

For developing survey questionnaires, we built on previous studies [27] that proposed a set of 10 indicators to assess social vulnerability to climate change: (i) demographically vulnerable groups, (ii) dependence on vulnerable resources and services, (iii) current household livelihood and income diversity, (iv) perceived alternative and supplementary livelihoods, (v) awareness of household vulnerability to climate hazards, (vi) access to and use of climate-related knowledge, (vii) formal and informal networks supporting climate hazard reduction and adaptation, (viii) ability of a community to reorganize, (ix) governance and leadership, and (*x*) equitable access to resources. The approach to assessing adaptive capacity used the following influential factors reported as key determinants of adaptive capacity: spatial configuration, environmental sensitivity, social cohesion, economic diversification, political-institutional structuring and living conditions, access and distribution of resources, technology, information and wealth; risk perceptions; social capital and community structure; and institutional frameworks that address climate change hazards [28, 29].

In this study, historical changes in climate variables (rainfall and temperature), physical information (importance of mangrove cover), and occurrence of extreme climatic events are taken as indicators of exposure. Loss of assets (land, crops, and equipment) due to climate-related disasters over recent years and income structure represent sensitivity for this study. Elements used to analyze adaptive capacity included human resource assets (awareness about climate change), social assets (membership in community-based organizations and participation in a development projects), financial assets (livelihood diversification), and physical assets (house quality and devices to access climate-related information).

The study adopted Klein and Nicholls's method to identify and classify adaptation options [30]: managed retreat, accommodation, and protection (Table 1).

### 2.3. Coastal Erosion Mapping

The mapping was constructed from Landsat images from 1973, 1986, 2000, and 2017, including Landsat 5 MSS, Landsat 7 TM, Landsat ETM, and Landsat 8.

These images were freely downloaded from the USGS website. The different coastlines were automatically extracted using ArcGIS software from an unsupervised classification. Having the coastlines mapped in four different years, a baseline is defined and is used as a reference for the calculation of the transects.  Table 2 shows the characteristics of the images used for this mapping.

The erosion modeling was done using the Digital Shoreline Analysis System (DSAS) [31]. The levels of vulnerability were rated on a scale of five (very high erosion, high erosion, low erosion, low accretion, and very strong accretion).

Two types of calculations were made:

- Net shoreline movement (NSM) reports a distance, not a rate. It is associated with the dates of only two shorelines. It reports the distance between the oldest and youngest shorelines for each transect [32]. This represents the total distance between the older and younger shorelines [33]. The overall change in shoreline position was estimated using NSM.

The endpoint rate (ERP) operator was used to calculate the rollback rate in m/year.

The end point rate (ERP) method is the distance on the transect between two shorelines, the most recent and the oldest, divided by the number of years between these shorelines [33, 34].

(1) $$\begin{array}{c} R=\frac{D}{T_{e}}, \end{array}$$

where *R* is the velocity in meters per year (m/year), *D* is the distance in meters, and Te is the time lapse between the oldest and newest coastlines (years).

EPR still works well even when only two coastlines are used to analyze evolution [31]. The parameters used to calculate change statistics included the shoreline change envelope (SCE), net shoreline movement (NSM), end point rate (EPR), linear regression rate (LRR), weighted linear regression (WLR), and least median square (LMS).

The precision of the shoreline definition is given in Table 3.

### 2.4. Field Data Collection Procedure

The study period was between June 2013 and February 2014. We followed a community-based vulnerability assessment approach [35], which assesses community vulnerability and its variables based on community perceptions. First, a literature review was done based on reports, research papers, documents, and other materials from various sources. A reconnaissance survey was conducted along the coastline of Manoka to become familiar with the study area, and nine settlements were selected for the study.

The subsequent field study consisted of focus group discussions/community meetings and household interviews in the sampled settlements. A total of 89 households participated in these discussions (Table 4). Household surveys were conducted to gather information on socioeconomic conditions, climatic phenomena, their effects on livelihoods, and the adaptation practices used to cope with them. The survey was conducted by using a semistructured questionnaire. Questions included in the questionnaire focused on climate change perceptions by the local population, activities and capita exposed to climate risks and response strategies pertaining to these, respondent's economic activities, sensitivity of activities and capita to climate risk, and access to and use of climate information.

Direct observation was carried out in the respondents' compound. Different adaptation strategies adopted by the people to cope with climate change were documented. These observations were utilized to triangulate the information gathered from the other sources.

Historical meteorological data needed for the research were collected at Douala Meteorological Station. Mainly temperature and rainfall data of the period from 1980 to 2010 were used. They were standardized and used to describe the climate profile of Manoka Island.

## 3. Results

### 3.1. Climate Profile of Manoka

**Figure 3:**
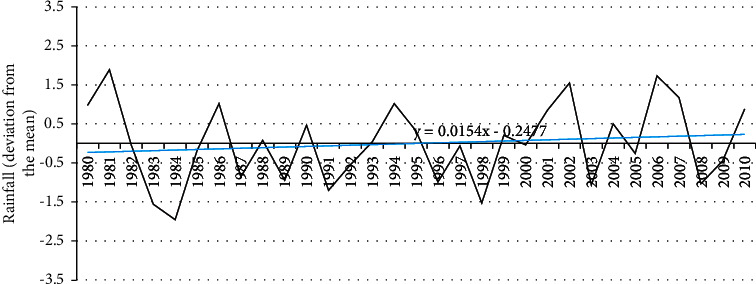
Rainfall data deviation from the mean in Manoka over the past decades. Source: Douala Meteorological Station.

**Figure 4:**
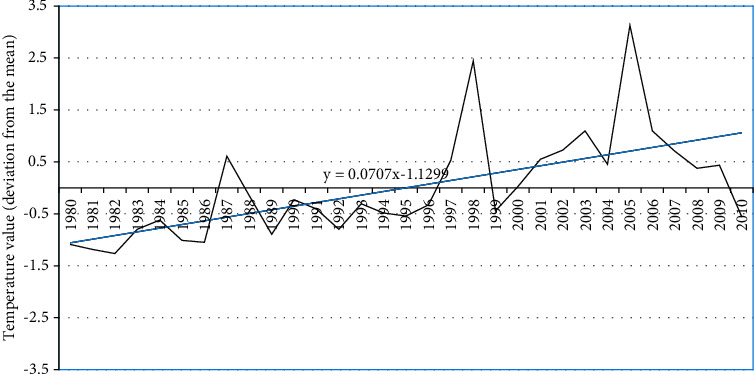
Temperature data deviation from the mean in Manoka over the past decades. Source: Douala Meteorological Station.

The historical records of climate data of the nearest meteorological station situated in Douala indicate that, over the past three decades, there have been only small variations in rainfall. Average monthly rainfall is 299.8 mm and average temperature is 27.2°C and the temperature trend has been significantly increasing over the past three decades (Figures 3 and 4).

### 3.2. Coastline Dynamics

**Figure 5:**
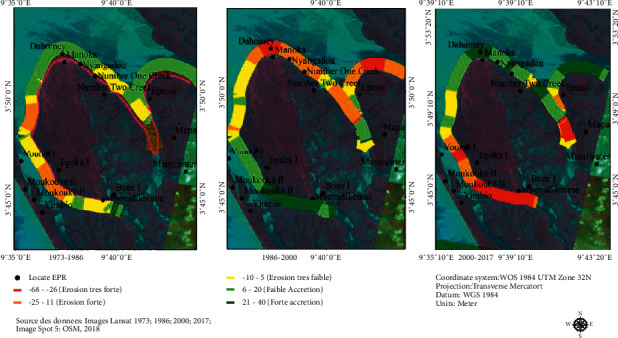
EPR erosion model around Manoka (m/year).

Coastal erosion is occurring around Manoka Island. Our analysis of the dynamics of the coastline between 1975 and 2017 using EPR shows setbacks of more than ±3 m/year. Erosion levels vary depending on the period (Figure 5).

Figure 5 shows the coastline dynamics around Manoka Island for the period from 1973 to 2017 (44 years). The treatment is done for three time scales: 1973–1986, 1986–2000, and 2000–2017.

During the period of 1973–1986, Manoka Island experienced very little erosion. The coastline was stable in the south toward the locality of Mbenadikoumé. However, at Epaka 1 a retreat of –1.8 m/year is observed along the coastline. At Dahomey and Nyangadou, there was accretion. The coastline progressed by +2.1 m/year, before stabilizing around Number One Creek and Number Two Creek.

During the period of 1986–2000, the island showed strong accretion on its southern side, with a coastline accretion estimated at ±4.5 m/year, toward the locality of Mbenadikoumé. To the north of the island, an accretion rate of ±2.3 m/year was measured between Epaka 1 and Youmé 1. North of this last locality, the coastline started to stabilize. To the north of Manoka Island, on the point facing the ocean, erosion has been observed. The retreat of the coastline is estimated at –2.4 m/year to both the east and west of Dahomey and –7 m/year in Dahomey. On the eastern shore of the island, a retreat of –1.9 m/year was measured at Nyangoudou, Nyangadou, and Number One Creek. Stability of the coastline is visible at Number Two Creek, followed by a retreat of 1.8 m/year and a slight accretion of 1.5 m/year.

Finally, during the period of 2000–2017, the entire southern part of the island was being eroded. The locality of Mbenadikoumé shows a retreat of about –5.1 m/year, followed by Youmé 1 (–4.2 m/year) and Epaka 1 with –2.3 m/year. The northern part was in accretion, with the peak at the locality of Dahomey (+4 m/year). The localities of Nyangadou (+2.3 m/year) and Number Two Creek (1.8 m/year) were also in accretion.

**Figure 6:**
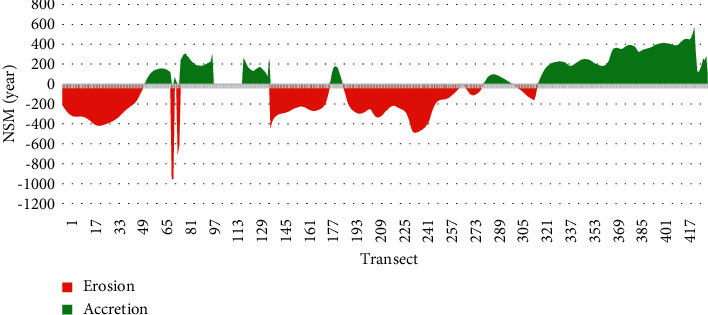
Dynamics of the coastline around Manoka Island between 1975 and 2017.

**Figure 7:**
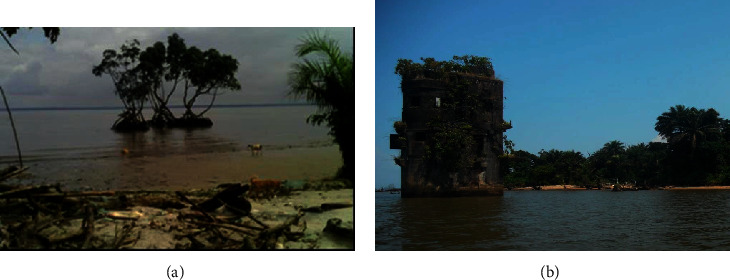
Sea advance accelerating coastal erosion. (a) Kwansi beach. (b) Dahomey beach.

The erosion profile of the area around Manoka Island recorded regression and accretions based on the net shoreline movement (NSM) in meters (Figures 6 and 7). This calculation is made on the basis of orthogonal transects generated using the DSAS® 4.7 software.

One typical example of coastal erosion threatening buildings and mangrove cover on this island is shown in Figure 7.  Figure 7(a) shows how the erosion is affecting coastal mangrove ecosystems and Figure 7(b) shows the location of a building that served as a prison during the German colonial period in the early 1900s. At that time, this building was on land, but it is currently surrounded by sea water.

### 3.3. Results of the Questionnaire Survey

#### 3.3.1. Importance of Climate-Related Stressors as Perceived by the Respondents

During the past decades, several extreme climate events have occurred in the area (Table 5). Some of the events such as coastal erosion, strong waves, and coastal storm surges were reported as being quite frequent, mostly occurring during periods of transition between the dry and the wet seasons. Table 3 summarizes the number of households reporting climate stressors. Rain storms, changes in season, floods, sea level rise, and coastal erosion are the five most frequent stressors perceived by local people.

#### 3.3.2. Household Perception of Climate-Induced Impacts

**Figure 8:**
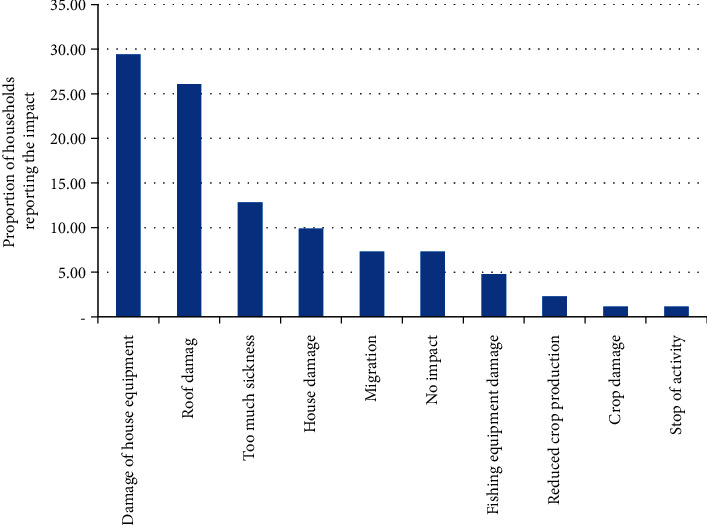
Impact of climate-related stressors as perceived by local people.

**Figure 9:**
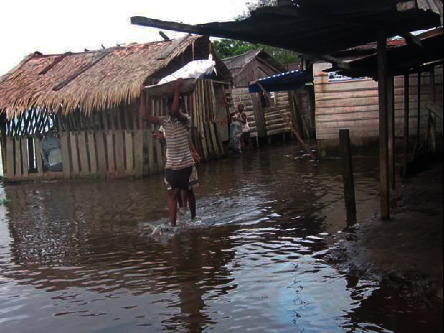
Case of flooding affecting houses in Kwansi settlement.

From community perception, infrastructure (housing) and health are the most affected sectors. Many beach houses, mostly built using wood materials, have become at risk of coastal erosion and house property damage was the impact most reported by the respondents (Figures 8 and 9).

#### 3.3.3. Respondents' Income Activities

**Figure 10:**
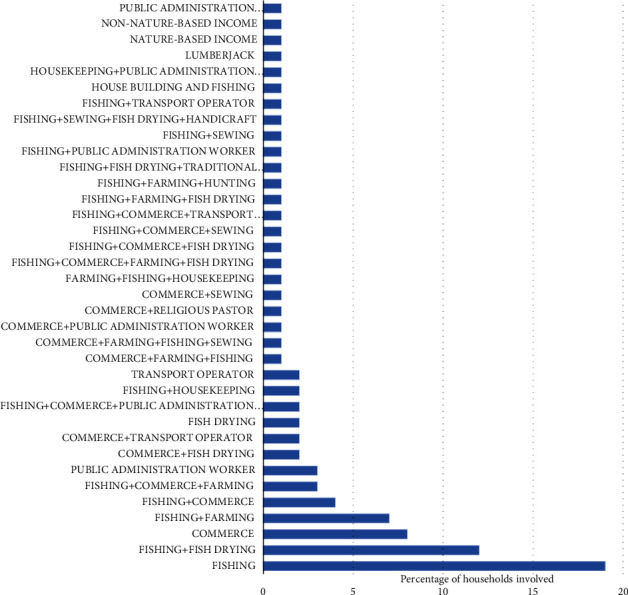
Livelihood diversification.

In Manoka island, local residents are highly dependent on fishing for their income but there is low livelihood diversification of households (Figure 10). Fishing occupies more than 80% of the population and is essentially dominated by Nigerian nationals who are the majority of the inhabitants. Apart from this activity, respondents were not involved in other income activities. Households not practicing fishing also relied in the majority on only one income source.

**Figure 11:**
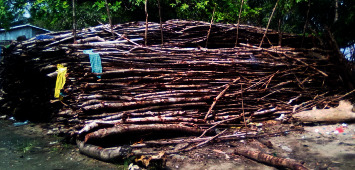
Mangrove wood piles for fish drying at Dahomey settlement.

Traditional drying techniques are not sustainable and rely heavily on mangrove wood. The resulting high demand for mangrove wood by fishermen is a serious problem in Manoka. Large areas of mangroves are cut down every year. In the Nyangadou fishermen's camp, for example, we can see piled up mangrove wood intended for smoking fish (Figure 11). The resulting deforestation and degradation of forest mangroves increase their vulnerability in different aspects, such as reduction of natural protection areas afforded by mangroves, reduction of food security from fishery products, and reduction of spawning areas essential for the reproduction of fish. Farming suffers from a lack of agricultural land. Soils are sandy and salty. On the island, we have only small farms that produce potatoes, yams, cassavas, maize, and pineapples. All these observations are indicative of the very poor livelihood diversification of families, making them more vulnerable.

#### 3.3.4. Housing

Because of the low level of income of the population and their perceptions^1^ (on the island, only 10% of the population are Cameroonian), houses built with permanent materials are rare. Indeed, 95% of the houses built on Manoka Island are made of temporary materials (wood). This increases their vulnerability to various natural hazards.

#### 3.3.5. Climate Change Awareness

Overall, the level of climate change awareness and access to climate information sources are still low among the communities. There is poor access of households to climate information sources, as reported by 65% of respondents. They have very little information obtained from locally operating NGOs, as well as TV and radio broadcasting. This is in most cases limited to sensitization messages on the adverse impacts of climate change, and weather information or adaptation/coping mechanisms are rarely mentioned.

However, based on their personal experiences, respondents generally agreed that the climate is changing. They see these changes as a threat, and they pointed out deforestation as the leading cause of climate change. Furthermore, there is a lack of knowledge of the United Nations Framework Convention on Climate Change (UNFCCC), the Kyoto Protocol, the Intergovernmental Panel on Climate Change (IPCC), and other significant climate change discussion platforms and mechanisms.

#### 3.3.6. Local Adaptation Strategies

**Figure 12:**
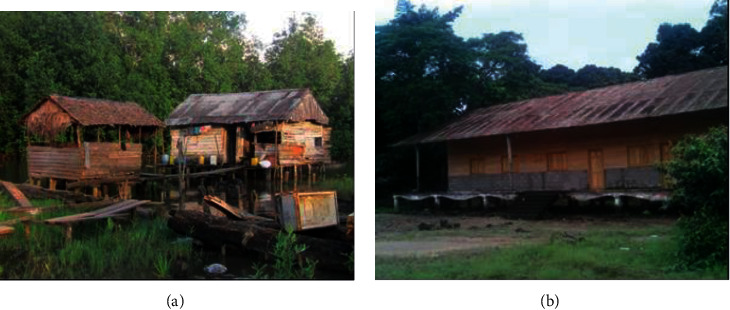
Houses elevated to adapt to flooding in Ngalamberi settlement 5 (a) and at the Manoka council headquarters (b).

The great majority of respondents did not use any long-term adaptation strategies. In most cases, these adaptation strategies are ineffective and inadequate to sustainably address the stressors faced (Table 6 and Figure 12).

## 4. Discussion

The vulnerability to climate risks investigated in this research conducted on Manoka Island focused on the three main components of vulnerability: exposure, sensitivity, and adaptive capacity.

### 4.1. Exposure to Climate Change in Mangrove Ecosystem

First, climatic risks were analyzed through historical data on the evolution of climatic parameters (rainfall and temperature) and the occurrence of extreme climatic events, which are all taken as exposure indicators.

The rainfall and temperature profile of Manoka is in line with the overall climate profile of Cameroon and is characterized by increasing temperature and decreasing rainfall patterns [33]. Another factor of exposure is mangrove cover, which has been analyzed by other researchers for the Manoka district. As is the case in most coastal areas of Cameroon, research conducted using a diachronic analysis of Landsat images and field surveys showed that the mangroves of Mabe in the Manoka district (Littoral Cameroon) have recorded a total degradation of 13% in 28 years (103 ha), representing an annual loss of 0.48% [13]. Coastal erosion similar to that observed along the coastline of Manoka Island has also been documented along other coasts of Central Africa, in particular in Gabon, as a driver of mangrove loss. The rapid erosion of the Pointe-Noire and Libreville coasts in the Republics of Congo and Gabon, respectively, has been reported in previous studies [34]. The mangrove belt has long been well documented as being protective against shoreline erosion because it reduces wave energy [35]. Hence, the more the mangrove belt is fragmented, the more the coastal area is exposed to the effects of climate change. The best approach to coastal erosion management, therefore, involves taking climate change into account.

### 4.2. Sensitivity to Climate Change in Mangrove Ecosystems

Climate change sensitivity has resulted in loss of assets (land, crops, and equipment) due to climate-related disasters in recent years, with damage to houses reported by the majority of respondents in this study. A similar situation was reported by Munji et al. [19] in the Limbé coastal areas of Cameroon. In flood-prone areas of Pakistan, higher vulnerability of the health, water, and land holding sectors was also reported [36].

This sensitivity of the study area to climate change has been exacerbated by the local population's income structure, characterized by high dependence on climate-dependent activities, especially fishing. To preserve and process the fish, only mangrove wood is used for smoking, which puts greater pressure on the mangrove, which is being destroyed to meet the firewood demand of the local residents as well as the inhabitants of the city of Douala. Mangrove destruction increases coastal erosion and flooding.

Regarding flooding, the manifestation of this hazard has been profiled over the entire African Atlantic coast [37]. Studies have reported the various modifications of the coastline linked to the aggregate effect of climate change and extreme events [38]. Previous studies in the mangrove settlements in Cameroon reported that floods have had an impact on the ecosystem by bringing saltwater inland, which has considerably degraded these environments and facilitated access to mangrove trees that were previously inaccessible. They have therefore led to their excessive exploitation [19, 39]. Such overexploitation of mangroves and forests leads to greenhouse gas (GHG) emissions and the loss of biodiversity of this landscape where many ecotones are founded.

### 4.3. Climate Change Adaptation in Mangrove Ecosystems

Adaptation constitutes the last aspect of the vulnerability assessment in this study. This capacity relates to the ability of local populations to cope with the various climatic hazards and the means at their disposal to do so.

Items used to analyze adaptive capacity included human resource assets and social assets (belonging to a community-based organization, existing support from a development project), financial assets (index of diversification of livelihoods), and physical assets (quality of the house and access to climate-related information). Overall it appeared that low-income diversity is a form of vulnerability in Manoka, as, on this island, 80% of the population depends mainly on fishing. This livelihood profile of the Manoka Island population is the reality of most coastal communities in Africa, as reported by previous studies [40]. Access to credit and educational resources and to training opportunities around climate change has been very limited on the island. As a result, the awareness of the population about climate change and its effects on social and ecological systems remains poor.

Local response initiatives are limited to constructing buildings on stilts, building overhead bridges, and using car wheels to counter the advancement of sea water inland. Some relocation of buildings is also observed. These strategies are generally ineffective and neither reduce the harmful effects of hazards nor limit their occurrence. Lessons learned from previous studies reports increased susceptibility of agriculture in the context of climate change. In Pakistan (Fahad and Jing, Fahad and Wang, and Fahad and Wang), studies revealed that farmers adopted various adaptation measures including change in crop type or variety and change in fertilizer and farming practices, and about 30% of the farmers accepted the concept of crop insurance as the mechanism for disaster risk reduction [41–43].

Restoration of mangroves has not yet been prioritized here as an adaptation strategy by communities. In the context where the vulnerability of the population and their ecosystems are considered as serious threats, bringing mangroves back can be an important step toward increasing the resilience of mangrove communities. Such restoration initiatives will comprise important activities that aim to combine both mitigation and climate change adaptation in this fragile ecosystem.

More generally, the implementation of poverty alleviation measures is also of importance. Studies in China have reported that sustainable livelihood was positively correlated with poverty alleviation measures, as well as natural and social capital [44].

## 5. Conclusion

This study aimed to investigate climate change vulnerability and adaptation on Manoka Island. The study used existing vulnerability frameworks, combined with focus group discussions and surveys with 89 households in nine settlements. Variables of exposure and sensitivity to climate risks, impacts on coastal communities, and local adaption strategies were described and assessed based on community perceptions and biophysical evidence.

The results indicate that the population on Manoka Island is exposed to climate change as shown by the increasing temperature and irregular rainfall patterns. Average annual values deviation from the mean was between −1.9 and +1.8 mm for rainfall and between −1.2 and + 3.12 for temperature. The respondents reported a series of extreme events that occurred on the island. The number of households perceiving seasonal variability, flood, and rain storm was higher. An erosion model shows that, between 1975 and 2017, average setbacks of the coastline of more than ±3 m/year, with varied erosion levels depending on the period and location, were recorded. The effect of climate stressors was perceived by the population as affecting more the infrastructure (housing) and the health sector. The reported high dependence of households on fishing for income, their overall low livelihood diversification, and their poor access to climate information reported by 65% of respondents portray their poor adaptive capacity. The adaptation strategies used are both reactive and preventive and are in most cases not cost-effective. They include constructing buildings on stilts and using car wheels to counter the advancement of seawater inland.

Our research concludes that households on Manoka Island are vulnerable to the impacts of climate change. Income diversification, planning of settlements, mangrove afforestation and climate education, the development of sustainable supply chains for wood fuel, the facilitation of the adoption of sustainable fish smoking devices, and, more broadly, the implementation of poverty alleviation measures are the main pathways for adaptation planning in this area. Mangrove restoration in particular will be an important opportunity to synergize mitigation and adaptation in this fragile ecosystem. It is therefore important to implement community development and an awareness program about climate change vulnerability which will help equip households with adequate facilities for adaptation. This study provides insights and background information that will be useful to policymakers, governmental agencies, and research-development organizations for adaptation planning in this area.

## Figures and Tables

**Table 1:** Framework for assessing adaptation strategies and options [30].

Strategy	Options
Managed retreat	No development in susceptible areas Conditional phased-out development Relocation projects

Accommodation	Modification of land use Modification of building styles and codes Strict regulation of hazard zones Hazard insurance

Protection	*Hard structural options*: Dikes, levees, and floodwalls Seawalls, revetments, and bulkheads Detached breakwaters Floodgates and tidal barriers Saltwater intrusion barriers *Soft structural options*: Periodic beach nourishment Dune/wetland restoration and creation

**Table 2:** Characteristics of images used.

Data (year/month/day)	Path and Row	Resolution	Code (bits)	Capteurs	Season	Purpose
1973/01/06	LM02_L1TP_200 057	60 m, resampled at 30 m	8	Mss	Dry season	Extraction of coast line
1986/12/12	LT05_L1TP_187057	30 m	8	Landsat 7 (TM)	Dry season	
2000/11/6	LE7 186 057	30 m	8	Landsat ETM	Dry season	
2017/12/20	LC08 187 057	30/15 m	16	Landsat 8	Dry season	

**Table 3:** Precision on shoreline definition.

Error margin			
Years	1973–1986	1986–2000	2000–2017
RMS error	0, 1	0, 1	—
Measurement error	32	42	48
Pixel error	2	4	3
Annual error EPR: ECI	0, 7–1, 3		

**Table 4:** Number of household members sampled per village.

Settlements	Location		Total population	Total number of households	Number of households interviewed
	N	E			
Nyangado	3.85822^o^	9.63938^o^	799	192	21
Kwensi	3.86438°	9.63046°	609	124	15
Plateau	3.86095°	9.62453°	398	85	22
Dahomey	3.86773^o^	9.62182°	734	161	13
Sandjè	3.84971^o^	9.60732°	212	48	5
Kombo Epacka	3.77175°	9.61323°	174	35	3
Mbengue Dikoumè	3.74794°	9.65642°	127	46	10
Number One	3.85075°	9.64710°			
Ngalamberi	3.79388°	9.69438°	318	68	
Total			3371	759	89

**Table 5:** Number of households reporting climate-related threats.

Climate stressors	Number of households perceiving		Number of households not perceiving		Number of households undecided	
	Number	Percentage	Number	Percentage	Number	Percentage
Seasonal variability	**64**	71.91	20	22.47	5	5.62
Cooler weather	15	16.85	62	69.66	12	13.48
Drought	14	15.73	69	77.53	6	6.74
Flood	**51**	57.30	33	37.08	5	5.62
Mudslide	8	8.99	72	80.90	9	10.11
Rainstorm	**76**	85.39	8	8.99	5	5.62
Saltwater intrusion	42	47.19	41	46.07	6	6.74
Sea level rise	**47**	52.81	37	41.57	5	5.62
Warmer weather	13	14.61	66	74.16	10	11.24

Bold values represent the climate stressors most reported by respondents.

**Table 6:** Climate change manifestation and adaptation initiatives.

Climate change manifestation experienced	Coastal adaptation		
	Protect	Accommodate	Retreat
Irregular rainfall/changes in seasons	—	(i) Resow crops when damage occurs	—

Sea level rise	(i) Use of sand-filled bags	(i) Take care of fishing equipment	—

Flooding	(i) Use of sand-filled bags	(i) Farm abandonment (ii) Change of crops (iii) Switch from farming to nonfarm activities (iv) Resow crops when damage occurs (v) House elevation (construction on stilts)	(i) Change of settlement (Kombo Epaka)
Rain storms	—	Reinforce roof protection Make new roof in cases of damage	—

Coastal erosion	(i) Use of sand-filled bags	Build new house in cases of damage House elevation	—

Mud slides	—		Change of settlement
Salt water intrusion	—	Use of sand-filled bags against flooding	—
Salt water corrosion	—	—	—
Rising temperature	—	—	—

## Data Availability

Readers can access the data supporting the conclusions of this study upon request to the corresponding author.
